# iTRAQ-based proteomic analysis to identify the molecular mechanism of Zhibai Dihuang Granule in the Yin-deficiency-heat syndrome rats

**DOI:** 10.1186/s13020-017-0160-y

**Published:** 2018-01-08

**Authors:** Chang-Ming Liu, Jing Chen, Su Yang, Ting-Ting Jiang, Zhong-Liang Chen, Hui-Hui Tu, Lian-Gen Mao, Yu-Ting Hu, Lin Gan, Zhong-Jie Li, Ji-Cheng Li

**Affiliations:** 1 Institute of Cell Biology, Zhejiang University, Hangzhou, People’s Republic of China; 2 South China University of Technology School of Medicine, Guangzhou, People’s Republic of China

**Keywords:** Herbal medicine, Zhibai Dihuang Granule, TCM, Proteomic analysis, Coagulation, Complement

## Abstract

**Background:**

Zhibai Dihuang Granule (ZDG) is a traditional Chinese medicine which has been used to treat Yin-deficiency-heat (YDH) syndrome for thousands of years in China. However, little work has been conducted to explore the molecular mechanism of ZDG in YDH syndrome, and the processes of YDH syndrome prevention and treatment have been developed slowly. The present study was aimed to explore the therapeutic mechanism of ZDG on YDH syndrome.

**Methods:**

The YDH syndrome rats were induced by hot Chinese herbs, then treated by ZDG orally for 1 week. Body weight was measured every 2 days. After sacrifice, blood samples were collected and the thymus, adrenal glands, spleen, and liver were immediately removed and weighed. iTRAQ-based proteomics approach was applied to explore the serum protein alterations with the treatment of ZDG, and to investigate the underlying mechanism of ZDG in treating YDH syndrome.

**Results:**

The body weights of YDH syndrome rats were significantly decreased compared with control group, and increased in ZDG treated rats. The relative weights of thymus in YDH syndrome rats were increased compared with the control rats, and significantly decreased in after ZDG treatment. In the proteomic analyses, seventy-one proteins were differentially expressed in the YDH syndrome group and the ZDG treated group, including 10 up-regulated and 61 down-regulated proteins. Gene ontology analysis revealed that the differentially expressed proteins were mostly related to immune response, and pathway enrichment analysis showed that these proteins were enriched in coagulation and complement cascades. Enzyme-linked immunosorbent assay was performed to detect the protein levels in coagulation and complement cascades, and the results showed that complement component 5 levels were significantly increased, while fibrinogen gamma chain levels were significantly decreased in the ZDG treated group.

**Conclusions:**

We found that ZDG treatment could lead to proteins alteration in immune response, especially in coagulation and complement cascades. ZDG can up-regulate the proteins in the complement cascade to eliminate pathogens, and down-regulate the proteins in the coagulation cascade to suppress inflammation. Our study provides experimental basis to understand the therapeutic mechanism of ZDG and revealed that ZDG can regulate coagulation and complement cascades in treating YDH syndrome.

**Electronic supplementary material:**

The online version of this article (10.1186/s13020-017-0160-y) contains supplementary material, which is available to authorized users.

## Background

Yin-deficiency-heat (YDH) syndrome is a common sub-health status in traditional Chinese medicine (TCM) characterized by fatigue, emaciation, five center (the palms, soles, and chest) heat, dry mouth, and tidal fever. If left untreated, YDH syndrome may develop into disease states, such as recurrent oral-ulcer, swollen gums and throat. YDH syndrome frequently occurs in individuals with yin-deficiency constitution, one of the most common pathological constitution in general population [1]. It is usually caused by long-term psychological stress, so it prevails especially among white collar workers and college students [2]. It has been reported that the incidence of YDH syndrome is significantly higher in individuals aged 15–34 than that in other age groups [1]. With the quickening pace of modern life and the increasing occupational stress, YDH syndrome is presenting a great challenge in China.

However, as a sub-health status, the appropriate conventional medicine to treat YDH syndrome is lacking. In TCM theory, the mechanism of YDH syndrome is considered as the deficiency of body fluid, especially in the mucous epithelium. It leads to the deterioration of moistening function, which finally result in the hyperactivity of internal heat in the body. Zhibai Dihuang Granule (ZDG), a classic traditional herbal medicine characterized by the function of nourishing Yin and suppressing internal heat, is commonly used to treat YDH syndrome clinically. ZDG is made from *Cornus officinalis*, *Rehmannia glutinosa*, *Dioscorea oppositifolia*, *Phellodendron amurense*, *Anemarrhena asphodeloides*, *Paeonia suffruticosa*, *Alisma plantago*-*aquatica* and *Poria cocos* [3]. *Anemarrhena asphodeloides* possesses the function of clearing away heat, nourishing Yin and moistening dryness. *Phellodendron amurense* is commonly used for purging pathogenic fire and expelling dampness. *Rehmannia glutinosa* possesses the effects of kidney-nourishing and essence-enriching. *Cornus officinalis* tonifies the liver and kidney, and *Dioscorea oppositifolia* invigorate spleen. *Paeonia suffruticosa* and *Alisma plantago*-*aquatica* display the activities of eliminating the internal heat [4]. The combination of these herbs may enhance the therapeutic effect on YDH syndrome. Currently, ZDG has been used not only in YDH syndrome management, but also to treat the concomitant symptoms of other diseases such as, diabetic nephropathy [5] and apoptosis of renal tubular cells [3]. However, owning to the diversity of the ingredients, and the complexity of the interaction between ZDG and the human body, the molecular mechanisms of therapeutic effects of ZDG are poorly understood. Furthermore, herbal medicine differs from the modern Western medicine in substance, methodology and philosophy [6], which impede Western countries from recognizing and accepting the therapeutic effects of the herbal medicine. Thus, there is an urgent need to reveal the therapeutic mechanism of ZDG on YDH syndrome.

Unlike the conventional medicine, herbal medicine usually treats patients in a holistic manner. As systems biology explores the complicated interactions among biological system components [7], it offers significant advantages to study the specific symptoms in TCM and the herbal medicine’s mechanism of action. Proteomics, one of the important part of systems biology, has developed to be a powerful tool to study protein changes in physiological conditions, illness, and the response to outside stimuli [8]. Proteomics provides systematic quantitative and qualitative mapping of the whole proteome in tissue, cultured cells and blood, and identify altered proteins as potential drug targets or biomarkers. Accordingly, by analyzing protein alterations before and after TCM treatment, the mechanism of action of TCM remedies can be explained and fully understood. In this study, iTRAQ-coupled 2D LC–MS/MS was used to explore alterations in serum protein levels after ZDG treatment. Furthermore, a series of bioinformatics approaches were applied to explore the therapeutic mechanism of ZDG.

## Methods

The minimum standards of reporting checklist (Additional file 1) contains details of the experimental design, statistics, and resources used in this study.

### Herbal medicine and animal experiments

The Chinese herbs such as, Fuzi (*Aconitum carmichaeli*, harvested in Sichuan province), Ganjiang (*Zingiber officinale Roscoe*, harvested in Guangdong province), and Rougui (*Cinnamomum cassia Presl*, harvested in Sichuan province), which are characterized by pungent and hot nature, were used to induce YDH syndrome in animal models [9–11]. Briefly, dried Fuzi, Ganjiang and Rougui (600 g each) were immersed in 4.5 L distilled water for 0.5 h. Then, the herbs were boiled with high heat, followed by simmering with gentle heat three times (25 min for the first time, 30 min for the second time, and 40 min for the third time). Finally, the extracts were merged together, then filtered, and concentrated to 2 g/mL. ZDG (batch no. 161204, each bottle containing 200 granules, 1.7 g for 10 granules), purchased from Zhongjing Wanxi Pharmaceuticals Ltd. Co. (Nanyang, China), was ground to a fine powder with a mortar and pestle and then dissolved in distilled water at a concentration of 0.57 g/mL. Female Sprague–Dawley rats (180–220 g) were purchased from the Experimental Animal Center of Zhejiang Province [License No. SCXK (Zhe) 2014-0001]. The rats were raised at a temperature-controlled (21–23 °C) and 12 h light/dark cycle room with free access to standard rat diet and water. All rats were acclimatized to the environment for 1 week before the experiments. The rats were randomly divided into the control group (N = 24), the YDH syndrome group (N = 20) and the ZDG treated group (N = 20). The rats in the control group were given sterile saline solution (2 mL/100 g) via gavage, and the rats in the YDH syndrome group and the ZDG treated group were given equal amount of Chinese herbal decoction via gavage for 2 weeks. On day 14, rats in the ZDG treated group (N = 20) were given ZDG (8.64 g/kg/day, via gavage) for 7 days, and rats in the YDH syndrome group and the control group were given equal amount of sterile saline solution for 7 days. The body weight of rats in each group was weighed every 5 days throughout the experiment. All rats were sacrificed at the end of the third week, and the blood samples were collected in the vacutainer tubes, and then clotted at room temperature for 1 h, followed by the centrifugation at 1500×*g* for 10 min at 4 °C to separate serum. The serum was aliquoted immediately in sterile centrifuge tubes and stored at − 80 °C. The thymus, adrenal glands, spleen, and liver were immediately removed and weighed. The experimental procedures were approved by the Zhejiang University Institutional Animal Care and Use Committee (China) and performed in compliance with the Guide for the Care and Use of Laboratory Animals, National Research Council (US) Institute for Laboratory Animal Research, 1996.

### iTRAQ-2D LC–MS/MS based proteomic analysis

#### Protein extraction

Serum samples from three group (18 rats per group) were subjected to protein extraction. In each sample, high abundant proteins albumin and IgG were removed using Pierce™ Albumin/IgG Removal Kit. Protein concentration was determined with 2-D Quant kit (GE Healthcare, Chicago, USA) according to the manufacturer’s instructions.

#### Trypsin digestion

The protein sample (100 μg) was reduced with 10 mM DTT (Sigma, St. Louis, MO, USA) for 1 h at 37 °C and alkylated at room temperature with 20 mM IAA (Sigma, St. Louis, MO, USA) for 45 min. Finally, trypsin was added with the ratio of protein:trypsin = 50:1 for the first digestion overnight and with the ratio of protein:trypsin = 100:1 for the second digestion for 4 h.

#### iTRAQ labeling

After digestion with trypsin, the peptides were desalted by using Strata X C18 SPE column and vacuum-dried. Then, the peptides were reconstituted in 0.5 M TEAB and processed for iTRAQ labeling according to the manufacturer’s protocol. Briefly, nine samples (three biological replicates per group) were labeled with the iTRAQ tags as control group (113 tags), YDH syndrome group (114 tags), and ZDG treated group (116 tags), and incubated at room temperature for 2 h. The labeled samples were then pooled and dried by vacuum centrifuging.

#### Strong cation exchange (SCX) fractionation

The pooled samples were subjected to Agilent 300 Extend C18 column (5 μm particles, 4.6 mm ID, 250 mm length, Phenomenex, CA, USA) for fractionation. Briefly, the samples were re-suspended with buffer A (25 mM NaH_2_PO_4_in 25% ACN, pH 2.6) and loaded onto the SCX column. The samples were then eluted with a gradient of buffer A at the flow rate of 1 mL/min for 10 min, 5–65% buffer B (25 mM NaH_2_PO_4_, 1 M KCl in 25% ACN, pH 2.6) for 11 min, and 65–100% buffer B for 1 min. The eluted peptides were combined into 18 fractions and dried by vacuum centrifuging.

#### LC–MS/MS analysis

The fractions were then subjected to a reversed-phase pre-column (Acclaim PepMap 100, Thermo Fisher Scientific, CA, USA) on an EASY-nLC 1000 UPLC system. Briefly, the fractions were re-suspended in buffer A (0.1% FA in 2% ACN) and loaded onto the column at 6 μL/min for 5 min. Then, the fractions were then eluted with 6–22% buffer B (0.1% FA in 98% ACN) for 26 min, 22–35% buffer B for 8 min, followed by a 3-min linear gradient to 80%, then holding at 80% for 3 min at a constant flow rate of 400 nL/min.

The eluted peptides were then subjected to NSI source followed by tandem mass spectrometry (MS/MS) in Q Exactive™ plus (ThermoFisher Scientific, CA, USA). The intact peptides were detected at a resolution of 70,000 in the Orbitrap. The peptides with normalized collision energy (NCE) setting of 30 were selected for MS/MS, and ion fragments were detected at a resolution of 17,500 in the Orbitrap. A data-dependent procedure that alternated between one MS scan followed by 20 MS/MS scans was applied for the top 20 precursor ions above a threshold ion count of 10,000 in the MS survey scan with 30.0 s dynamic exclusion. The electrospray voltage was set as 2.0 kV. Automatic gain control (AGC) was applied to prevent overfilling of the Orbitrap, and 5e^4^ ions were accumulated for generation of MS/MS spectra. For MS scans, the m/z scan range was 350–1800. Fixed first mass was set as 100 *m/z*. Each SCX fraction was analyzed twice.

#### Database search

The MS/MS data were searched against *Uniprot Rattus norvegicus* database by using Mascot search engine (v.2.3.0). Trypsin was set as cleavage enzyme allowing on more than 2 missing cleavages. For precursor ions, the mass error was set to 10 ppm, and for fragment ions, the mass error was set to 0.02 Da. Oxidation on Met was considered as variable modification, and carbamidomethylation on Cys was considered as fixed modification. Proteins with the false discovery rate (FDR) ≤ 1% were considered for further analysis. Protein ratios with the fold changes ≥ 1.20 or ≤ 0.83, and a *p* value less than 0.05 were considered significant.

### Bioinformatics analysis

The interaction gene networks of the identified proteins were analyzed by GeneMANIA (http://www.genemania.org/). The interaction networks of the identified proteins were analyzed by STRING (Search Tool for the Retrieval of Interacting Genes/Proteins) database (http://string-db.org/). The biological process, molecular function and cellular component were analyzed by searching gene ontology (GO) database (http://geneontology.org/). The pathway analysis was performed by Kyoto Encyclopedia of Genes and Genomes (KEGG) pathway database (http://www.genome.jp/kegg/mapper.html).

### Enzyme-linked immunosorbent assay (ELISA)

Based on the bioinformatics analysis and fold changes of differentially expressed proteins, we selected serum proteins involved in coagulation and complement cascades for ELISA validation. Rat C4b-binding protein alpha chain (C4bpa) ELISA kit (Cusabio, detection limit 39 ng/mL), rat complement fragment 5 (C5) ELISA kit (Cusabio, detection limit 2.34 ng/mL), and rat complement component 9 (C9) ELISA kit (Cusabio, detection limit 1.56 ng/mL) were proteins in the complement cascade. Rat coagulation factor VII (F7) ELISA kit (Cusabio, detection limit 0.195 ng/mL), rat fibrinogen alpha chain (Fga) ELISA kit (Cusabio, detection limit 0.025 μg/mL), rat fibrinogen beta chain (Fgb/Ab1-181/Ab1-216/Ac1-581) ELISA kit (Cusabio, detection limit 0.156 μg/mL), and rat von Willebrand Factor (vWF) ELISA kit (Cusabio, detection limit 0.078 ng/mL) were proteins in the coagulation cascade. ELISA was performed according to the manufacturer’s instructions.

### Statistical analysis

The experimental data was analyzed using the GraphPad Prism 5 (GraphPad Software Inc., USA). Comparison between two groups were performed using Mann–Whitney test, and multiple comparisons were performed using one-way ANOVA. The data were expressed as mean ± standard deviation (SD). p value less than 0.05 was considered statistically significant.

## Results

### ZDG increased the body weight in YDH syndrome rats

**Fig. 1:**
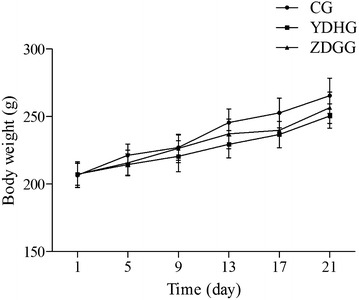
Effects of ZDG on body weight in YDH syndrome rats. Body weight was measured every 5 days. *YDHG YDH* syndrome group, *ZDGG* ZDG treated group, *CG* control group. The values are presented as the mean ± SD

Throughout the 14-day construction period of YDH syndrome animal model, rats in the YDH syndrome group and ZDG treated group had lower body weight than that in the control group (Fig. 1). At the end of the second week, the body weight in the YDH syndrome group and ZDG treated group were significantly decreased than the control group (*p* < 0.001, Table 1). During the 7-day ZDG treated period, the body weight of rats (256.50 ± 11.53, day 21) increased in the ZDG treated group, compared to the rats in the YDH syndrome group (250.45 ± 8.96, day 21, Table 1).

**Table 1:** Effect of ZDG on body weight in YDH syndrome rats

	CG		YDHG		ZDGG	
	Mean	SD	Mean	SD	Mean	SD
Day 1	206.50	9.16	207.26	8.21	207.15	9.48
Day 5	221.36	8.38	214.48**	7.83	215.68*	9.49
Day 9	227.00	9.47	220.62*	11.45	226.38	10.60
Day 13	245.60	9.99	229.45***	10.07	237.19**	10.97
Day 17	252.64	10.98	236.65***	9.83	239.82**	12.93
Day 21	265.40	13.10	250.45***	8.96	256.50*	11.53

*SD* standard deviation, *CG* control group, *YDHG* YDH syndrome group, *ZDGG* ZDG treated group

* Significant difference from the control group on the same day (Mann–Whitney U-test. * *p* < 0.05, ** *p* < 0.01, *** *p* < 0.001)

#### Effect of ZDG on the relative organ weight in YDH syndrome rats

**Fig. 2:**
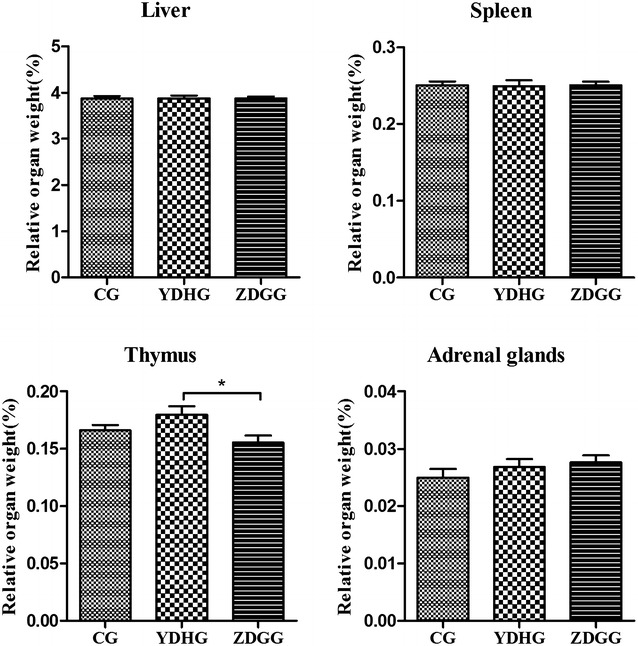
Comparison of relative organs weight in YDHG, ZDGG and CG. The values are presented as mean ± SD. Differences in each group were determined by using the Mann–Whitney U-test, and *p* < 0.05 indicates statistical significance. *Significant difference (*p* < 0.05). *CG* control group, *YDHG* YDH syndrome group, *ZDGG* ZDG treated group

At the end of animal experiments, the liver, spleen, thymus, and adrenal glands were immediately removed and relative weights of the organs in each group were calculated. The results indicated that the relative weights of liver, spleen and adrenal glands showed no significant differences in the three groups, while the relative weights of thymus in YDH syndrome rats were increased compared with the control rats, and significantly decreased (*p* = 0.017) compared with the ZDG treated rats (Fig. 2).

#### Effect of ZDG on the serum protein expression in YDH syndrome rats

**Fig. 3:**
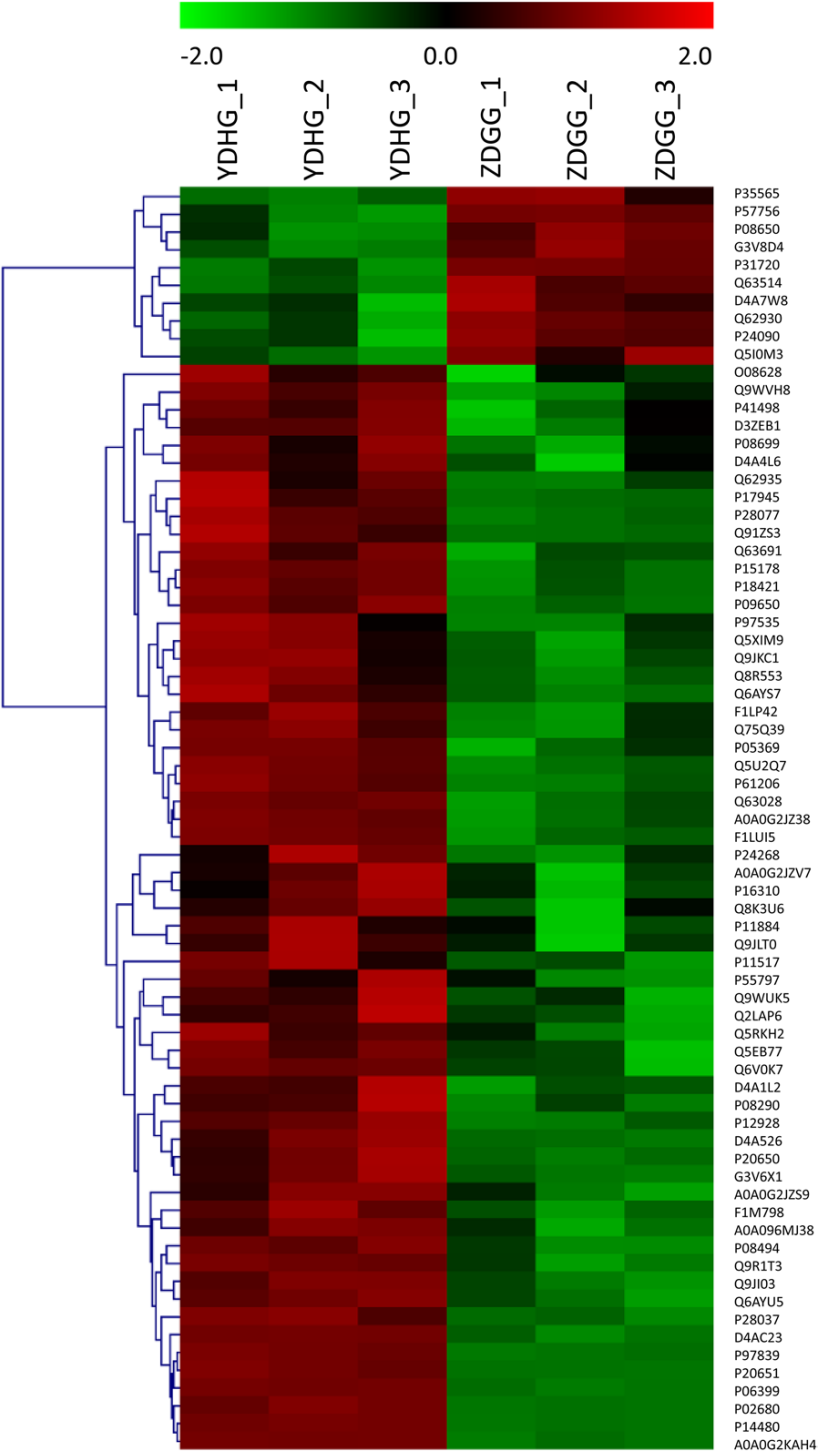
Heat map visualization of the differentially expressed proteins with the treatment of ZDG. Red, up-regulation; green, down-regulation

**Fig. 4:**
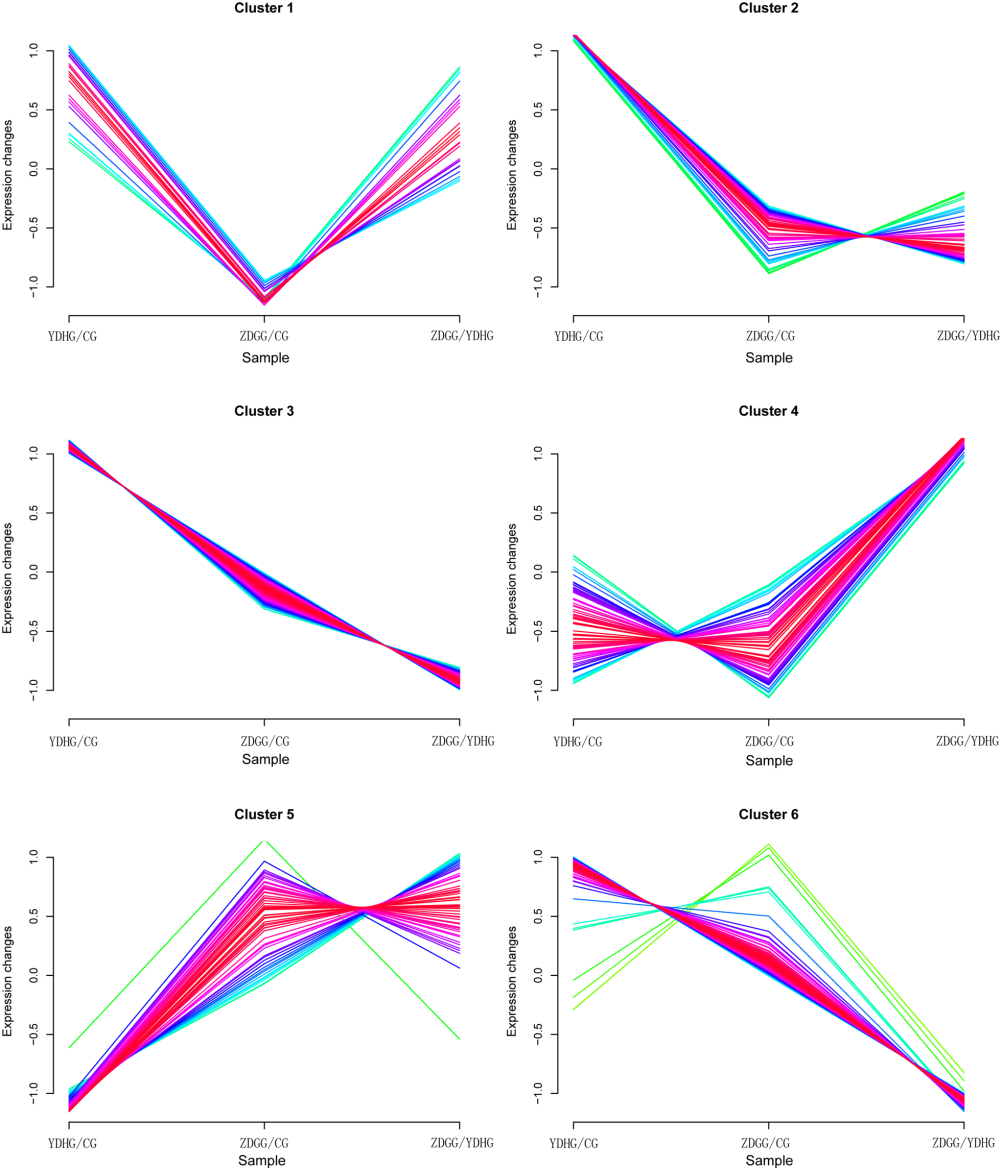
The expression clusters of the differentially expressed proteins in YDHG, ZDGG and CG. YDHG/CG, the protein ratio between YDH syndrome group and control group; ZDGG/YDHG, the protein ratio between ZDG treated group and YDH syndrome group; ZDGG/CG, the protein ratio between YDH syndrome group and control group

In the iTRAQ-2D LC–MS/MS analyses, a total of 1049 proteins were identified with three biological replicates, among which 997 proteins were quantified (see Additional file 2). Among the quantified proteins, 71 proteins (10 up-regulated and 61 down-regulated proteins) showed statistically significant changes (at least a 1.20-fold change and *p* < 0.05) in the YDH syndrome group and ZDG treated group (Fig. 3). According to the expression profiles of proteins in the YDH syndrome group, ZDG treated group and control group, we classified the differentially expressed proteins into 6 clusters (Fig. 4). The fold change and regulated type of the differentially expressed proteins were presented in Additional file 3.

**Fig. 5:**
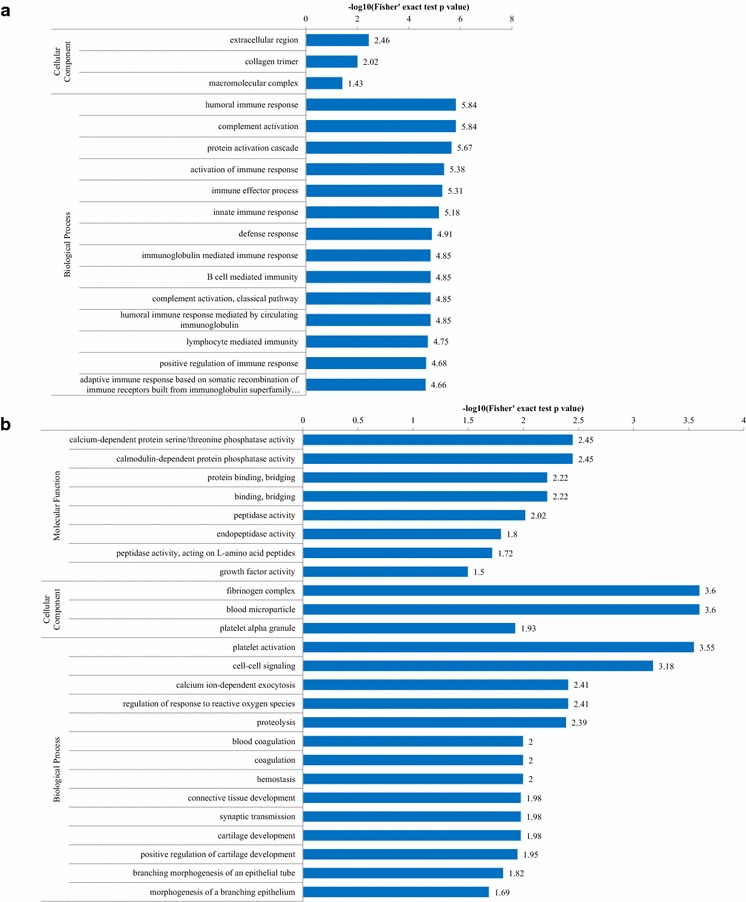
Bioinformatics data mining of the set of differentially expressed proteins with the treatment of ZDG. The GO terms are sorted by −log10 of the Fisher’s exact test *p* value, which indicates the enrichment significance of GO terms. **a** GO enrichment analysis of up-regulated proteins. **b** GO enrichment analysis of down-regulated proteins

**Fig. 6:**
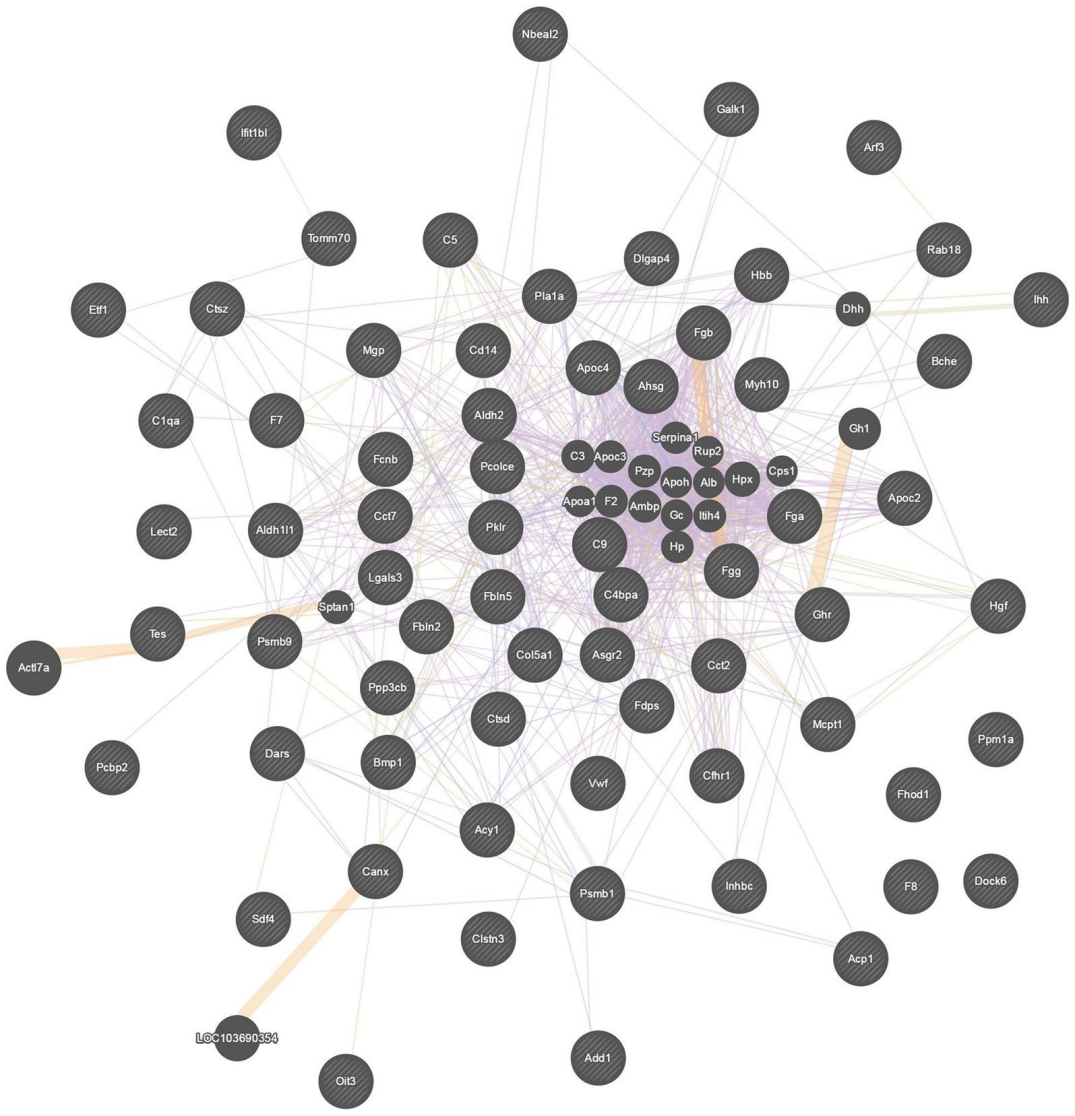
The interacted gene network of the differentially proteins analyzed by GenMANIA. Purple line, co-expression; orange line, predicted; blue line, co-localization; yellow line, shared protein domains

Among 10 up-regulated proteins in the ZDG treated group, the GO analysis indicated that most proteins were involved in humoral immune response (5 proteins), complement activation (5 proteins), activation of immune response (5 proteins), immune effector process (5 proteins), innate immune response (5 proteins), immunoglobulin mediated immune response (4 proteins), B cell mediated immunity (4 proteins), complement activation (classical pathway, 4 proteins), humoral immune response mediated by circulating immunoglobulin, lymphocyte mediated immunity (4 proteins), positive regulation of immune response (5 proteins), and adaptive immune response based on somatic recombination of immune receptors built from immunoglobulin superfamily domains (4 proteins), indicating that the up-regulated proteins mainly participated in immune response (Fig. 5a). Among the 61 down-regulated proteins, the GO analysis revealed that most proteins were associated with proteolysis (13 proteins), blood coagulation (6 proteins), coagulation (6 proteins), hemostasis (6 proteins), and platelet activation (5 proteins), revealing a great abundance in coagulation in down-regulated proteins (Fig. 5b). The interacted gene network analyzed by GenMANIA (http://genemania.org/) indicated that most genes encoding the differential proteins were co-expressed (Fig. 6).

**Fig. 7:**
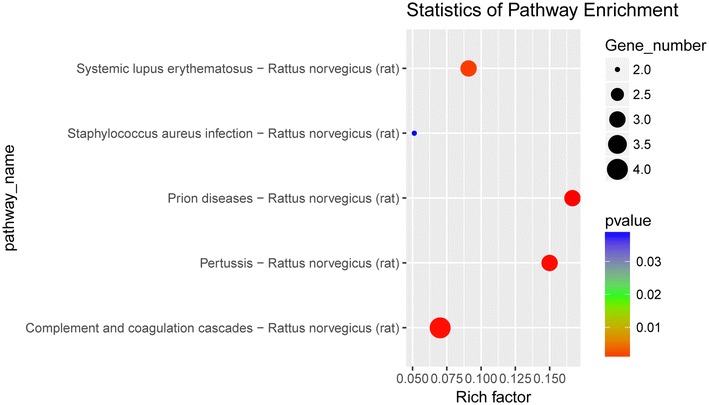
KEGG pathway analysis of the differentially expressed proteins with the treatment of ZDG. A two-tailed Fisher’s exact test was used to test the enrichment of the differentially expressed protein against all identified proteins, and enrichment of KEGG terms were presented in the heat map from low (green) to high (red)

**Fig. 8:**
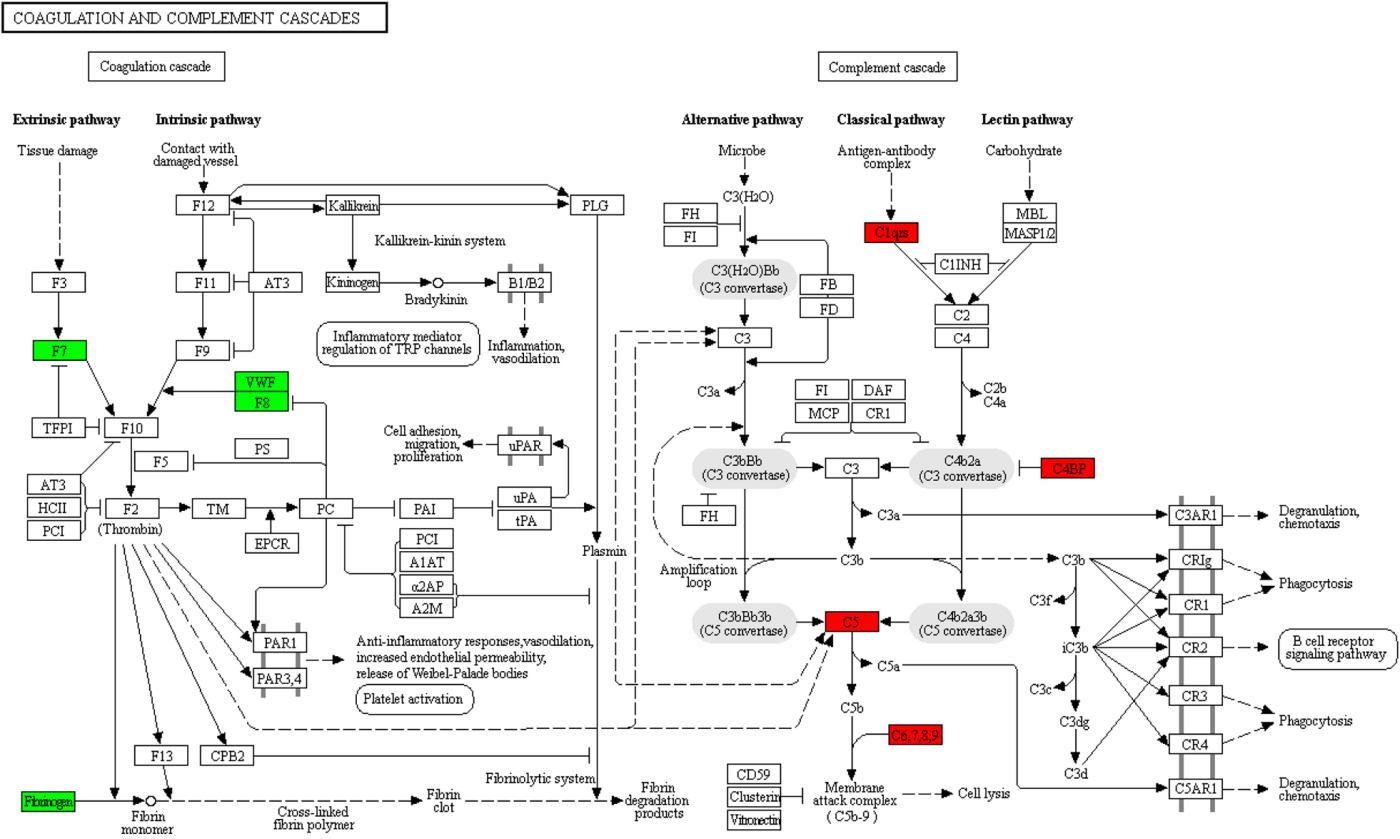
The differentially expressed proteins in coagulation and complement cascades. Red, up-regulated proteins; green, down-regulated proteins

KEGG pathway and STRING analyses showed that most differentially expressed proteins in the ZDG treated group were enriched in coagulation and complement cascades (Fig. 7). Besides, the proteins in coagulation cascades displayed down-regulation, while those in complement cascades displayed up-regulation (Fig. 8, Additional file 4).

#### Validation of proteins expression in coagulation and complement cascades

**Fig. 9:**
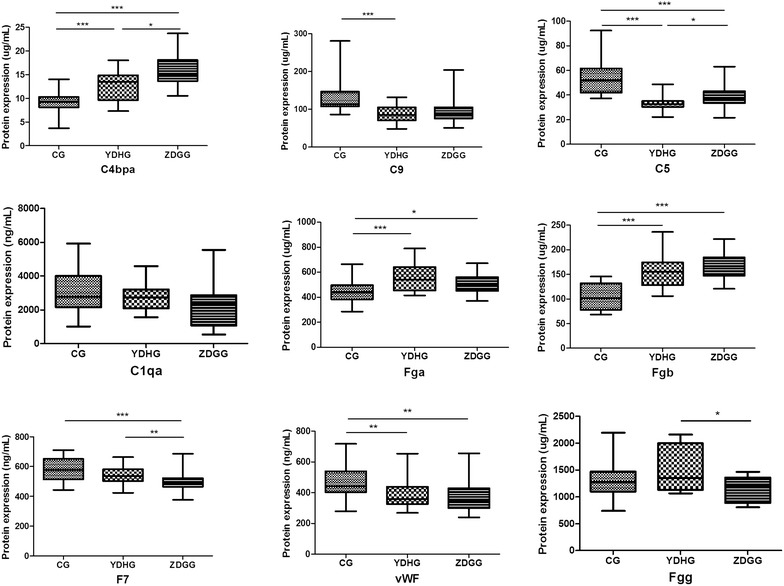
Verification of the differentially expressed proteins in coagulation and complement cascades by ELISA. Proteins expression were measured in the control group (n = 24), the YDH syndrome group (n = 20) and the ZDG treated group (n = 20). *p* values were calculated with the Mann–Whitney U-test, *p < 0.05, **p < 0.01, ***p < 0.001. *CG* control group, *YDHG* YDH syndrome group, *ZDGG* ZDG treated group

ELISA was performed to detect the serum expression of the proteins in coagulation and complement cascades. The results showed that serum expression of C4bpa and C5 levels were significantly increased in the ZDG treated group compared with the YDH syndrome group (*p* = 0.028, *p* = 0.018, respectively). C5 and C9 showed the trend of returning to normal after ZDG treatment. Serum expression of F7 and Fgg were significantly decreased in the ZDG treated group compared with the YDH syndrome group (*p* = 0.007, *p* = 0.033, respectively), and Fgg returned to normal. The levels of Fga, and vWF were lower in the ZDG treated group than the YDH syndrome group, and Fga was showed the trend of returning to normal after ZDG treatment, but no significant difference was observed (Fig. 9). In conclusion, serum expression of C5, C9, Fga, and Fgg were observed the trend of returning to normal condition in the ZDG treated group. The results revealed increased levels of proteins in complement cascade and decreased levels of proteins in coagulation cascade after ZDG treatment.

#### Quality control validation of MS data

The MS data validation is shown in supplementary figures. The mass error of all the identified peptides was checked, and the distribution of mass error was near zero and most of them were < 0.02 Da, indicating that the mass accuracy of the MS data fits the requirement. The length of most peptides was distributed between 8 and 16, which agree with the property of tryptic peptides. Pearson correlation analysis was used to estimate repeatability in three repeats of the MS data (see Additional file 5).

## Discussion

YDH syndrome is common in TCM practice. Although the mechanisms of YDH syndrome are still unclear, it is widely believed that the excessive consumption of Yin results in the pathological condition called “internal heat” in TCM theory. YDH syndrome has been demonstrated to be associated with depressed immunity and enhanced inflammation. Previous study revealed the decreased immunological substances [12] and increased inflammatory cytokines [13] in YDH constitution. Individuals with YDH syndrome present with five center (the palms, soles, and chest) heat, tidal fever, recurrent oral-ulcer, swollen gums and throat, which is closely related to the inflammatory reaction. Inflammation is an immune response characterized by the release of chemokines and cytokines [14]. Interestingly, TNF-α, IL-1β, and IL-6 levels have been shown to be up-regulated in YDH syndrome individuals [15], indicating that the inflammatory response could be enhanced in YDH syndrome. Thus, we hypothesized that YDH syndrome shares common biological basis with immune response and inflammation.

Herbal medicines have been widely used to manage and prevent diseases. ZDG is a well-known classic traditional herbal medicine to treat YDH syndrome. ZDG shares the similar ingredients with the Liuwei Dihuang Granule (LDG) herbal medicine, which has been reported to decrease the inflammatory cells in autoimmune encephalomyelitis [16]. However, few articles have reported the therapeutic mechanism of ZDG in treating YDH syndrome. In the present study, we found that 71 serum proteins were differentially expressed with ZDG treatment. GO analysis of these proteins revealed the enrichment of immune response in up-regulated proteins, and the enrichment of coagulation in down-regulated proteins. Both KEGG and STRING analyses indicated that the differentially expressed proteins after ZDG treatment were mainly involved in the coagulation and complement cascades pathway. Therefore, we hypothesized that ZDG can treat YDH syndrome by regulating proteins in the coagulation and complement cascades pathway.

The coagulation and complement cascades pathway is highly associated with immunity and enhanced inflammation. The complement system is a key sentinel of innate immunity, while the coagulation system serves as main actor in hemostasis. Both (coagulation and complement systems) belong to the “first line of defense” against injurious stimuli and invaders [17]. There is extensive cross-talk between inflammation and coagulation [18]. Inflammation induces activation of coagulation, and coagulation proteases modulate inflammation [17]. The extrinsic coagulation cascade is initiated by the combination of factor VII (F7) and the tissue factor (TF). The activated factor VII (F7a) activates both factor X (F10) and factor IX (F9), and the activated factor X (F10a) has pro-inflammatory properties [19]. However, in the intrinsic coagulation cascade, vWF prevents factor VIII (F8) from being activated, consequently inhibiting the activation of F9 and F10 [20]. F10a catalyzes prothrombin into thrombin, the key hydrolytic enzyme in the coagulation cascade, which induces up-regulation of various pro-inflammatory cytokines, including monocyte chemotacticprotein-1, IL-6, IL-8, and macrophage migration [20–22]. In our proteomics study, F7 and vWF were decreased after the ZDG treatment, indicating that ZDG can inhibit the enzymes in both extrinsic and intrinsic coagulation cascades. Fibrinogen, assembled by α-chain (Fga), β-chain (Fgb), and γ-chain (Fgg) participate in inflammatory response. Fibrin, the production of fibrinogen, regulates the generation of inflammatory cytokine in vivo [22, 23]. Fibrinogen was found to be down-regulated with the treatment of ZDG in the proteomics experiments, and the serum levels of Fga and Fgg were confirmed to be decreased by ELISA. Therefore, the repressed fibrinogen level by ZDG treatment could result in the suppression of inflammatory response.

Complement was initially thought to be the heat-sensitive fraction in human plasma which improves the antibodies in their capacity to eliminate pathogens. Activation of the complement cascade enhances the immune function. In classical pathway (CP), the recognition of pathogens occurs directly via contacting the pathogen-associated molecular patterns (PAMPs) by C1q, followed by activation of C1r and C1s [24]. C4 and C2 are subsequently cleaved by the activated C1s to form C4b2a [25]. As a C3 convertase, C4b2a cleaves C3 into the fragments C3a and C3b, the latter can be covalently bound to the pathogens via its exposed thioester [26]. When C3b reaches a certain amount on the surface of pathogens, the terminal pathway (TP) of complement is initiated. In TP, the C3 convertase C3bBb and C4b2a can interact with C3 to form C3bBb3b and C4b2a3b, both of which are C5 convertases. C5 is cleaved by these convertases to generate C5a and C5b, and the latter in combination with C6, C7, C8, and C9 form the membrane attack complex (MAC) [27]. Previous studies have demonstrated that the sublytic MAC can drive inflammation by activating NLRP3 inflammasome and triggering the release of cytokines IL-1β and IL-18 [28, 29]. Our results revealed the increased serum levels of C4bp, C5, and C9 after ZDG treatment, indicating that ZDG can enhance the activation of the complement cascade, and improve the ability to eliminate pathogens.

## Conclusions

In summary, treatment with ZDG significantly increased the protein expression in the complement cascade to promote the complement activation, and enhanced the ability to eliminate pathogens in immune process. Besides, ZDG also decreased the protein expression in the coagulation cascade to alleviate the inflammation. The results suggested that ZDG could treat YDH syndrome by regulating complement and coagulation cascades pathway.

## Additional files

**Additional file 1.** Minimum standards checklist.

**Additional file 2.** All proteins quantified by three biological replicates.

**Additional file 3.** Differentially expressed serum proteins between ZDGG and YDHG.

**Additional file 4.** STRING analyses of the differentially expressed proteins.

**Additional file 5.** Quality control validation of MS Data.

## Abbreviations

ZDG: Zhibai Dihuang Granule
YDH: Yin-deficiency-heat
GO: gene ontology
ELISA: enzyme-linked immunosorbent assay
KEGG: Kyoto Encyclopedia of Genes and Genomes
C4bpa: complement component 4 binding protein alpha
C5: complement component 5
C9: complement component 9
F7: coagulation factor VII
Fga: fibrinogen alpha chain
Fgg: fibrinogen gamma chain
vWF: von Willebrand Factor
TCM: traditional Chinese medicine
DTT: dithiothreitol
IAA: 3-indoleacrylic acid
iTRAQ: isobaric tags for relative and absolute quantification
SPE: solid phase extraction
SCX: strong cation exchange
ACN: acetonitrile
FA: formic acid
UPLC: ultra performance liquid chromatography
NCE: normalized collision energy
AGC: automatic gain control
LDG: Liuwei Dihuang Granule
TF: tissue factor
PAMPs: pathogen-associated molecular patterns
CP: classical pathway
TP: terminal pathway
MAC: membrane attack complex
NLRP3: NACHT, LRR and PYD domains-containing protein 3
SD: standard deviation
