# Supplementary material for: iTRAQ-based proteomic analysis to identify the molecular mechanism of Zhibai Dihuang Granule in the Yin-deficiency-heat syndrome rats
Source: Chin Med. 2018 Jan 8;13:2. doi: 10.1186/s13020-017-0160-y (PMC5759191; doi:10.1186/s13020-017-0160-y)
Supplement: Supplementary file 3 — Additional file 3. Differentially expressed serum proteins between ZDGG and YDHG. [file 13020_2017_160_MOESM3_ESM.docx]

**Table S1**. Differentially expressed serum proteins between ZDGG and YDHG.

| **Protein accession** | **Protein description** | **Fold change** | **Regulated Type** | **P value** |
| --- | --- | --- | --- | --- |
| Q62930 | Complement component C9 | 1.23 | Up | 0.0125 |
| P06399 | Fibrinogen alpha chain | 0.6 | Down | 0.0344 |
| P14480 | Fibrinogen beta chain | 0.48 | Down | 0.0376 |
| Q63514 | C4b-binding protein alpha chain | 1.27 | Up | 0.0118 |
| P24090 | Alpha-2-HS-glycoprotein | 1.22 | Up | 0.0015 |
| P02680 | Fibrinogen gamma chain | 0.5 | Down | 0.0293 |
| P31720 | Complement C1q subcomponent subunit A | 1.28 | Up | 0.0099 |
| P18421 | Proteasome subunit beta type-1 | 0.81 | Down | 0.0079 |
| Q8K3U6 | Coagulation factor VII | 0.77 | Down | 0.0292 |
| O08628 | Procollagen C-endopeptidase enhancer 1 | 0.83 | Down | 0.0165 |
| P11517 | RAT_ Hemoglobin subunit beta-2 | 0.75 | Down | 0.0427 |
| P97535 | Phospholipase A1 member A | 0.81 | Down | 0.0337 |
| Q5I0M3 | Complement component factor h-like 1 | 1.21 | Up | 0.0157 |
| D3ZEB1 | Protein F8 | 0.83 | Down | 0.0388 |
| P61206 | ADP-ribosylation factor 3 | 0.79 | Down | 0.0233 |
| P55797 | Apolipoprotein C-IV | 0.74 | Down | 0.0338 |
| P28077 | Proteasome subunit beta type-9 | 0.82 | Down | 0.0494 |
| A0A0G2JZV7 | RAT_ Uncharacterized protein | 0.77 | Down | 0.0140 |
| Q9WVH8 | Fibulin-5 | 0.83 | Down | 0.0161 |
| Q5XIM9 | T-complex protein 1 subunit beta | 0.81 | Down | 0.0059 |
| P24268 | Cathepsin D | 0.77 | Down | 0.0012 |
| Q62935 | von Willebrand factor (Fragment) | 0.83 | Down | 0.0029 |
| Q9WUK5 | Inhibin beta C chain | 0.74 | Down | 0.0121 |
| A0A0G2JZ38 | Protein Fhod1 | 0.78 | Down | 0.0122 |
| P41498 | Low molecular weight phosphotyrosine protein phosphatase | 0.83 | Down | 0.0291 |
| P15178 | "Aspartate--tRNA ligase, cytoplasmic " | 0.81 | Down | 0.0180 |
| G3V8D4 | Apolipoprotein C-II (Predicted) | 1.32 | Up | 0.0298 |
| A0A0G2JZS9 | RAT_ Uncharacterized protein | 0.71 | Down | 0.0047 |
| D4AC23 | Protein Cct7 | 0.63 | Down | 0.0444 |
| P17945 | Hepatocyte growth factor | 0.83 | Down | 0.0277 |
| Q9JLT0 | Myosin-10 | 0.76 | Down | 0.0062 |
| Q91ZS3 | 45 kDa calcium-binding protein | 0.82 | Down | 0.0221 |
| Q6V0K7 | Oncoprotein-induced transcript 3 protein | 0.73 | Down | 0.0310 |
| G3V6X1 | "Fibulin 2, isoform CRA_a " | 0.71 | Down | 0.0300 |
| Q5U2Q7 | Eukaryotic peptide chain release factor subunit 1 | 0.79 | Down | 0.0390 |
| Q6AYU5 | Poly(RC) binding protein 2 | 0.66 | Down | 0.0021 |
| D4A4L6 | RAT_ Uncharacterized protein | 0.83 | Down | 0.0487 |
| P12928 | Pyruvate kinase PKLR | 0.73 | Down | 0.0400 |
| P05369 | Farnesyl pyrophosphate synthase | 0.79 | Down | 0.0409 |
| F1LP42 | Hedgehog protein | 0.79 | Down | 0.0276 |
| F1LUI5 | Protein LOC100365438 | 0.77 | Down | 0.0344 |
| P08494 | Matrix Gla protein | 0.68 | Down | 0.0039 |
| P16310 | Growth hormone receptor | 0.77 | Down | 0.0377 |
| P57756 | Ficolin-2 | 1.41 | Up | 0.0049 |
| F1M798 | Metalloendopeptidase | 0.7 | Down | 0.0482 |
| P08650 | Complement C5 (Fragment) | 1.41 | Up | 0.0036 |
| D4A7W8 | Microfibrillar-associated protein 4 | 1.25 | Up | 0.0245 |
| Q63028 | Alpha-adducin | 0.78 | Down | 0.0368 |
| D4A1L2 | Protein Nbeal2 | 0.73 | Down | 0.0048 |
| Q8R553 | Calsyntenin-3 | 0.81 | Down | 0.0116 |
| Q2LAP6 | Testin | 0.74 | Down | 0.0498 |
| Q9JKC1 | Carboxylic ester hydrolase | 0.81 | Down | 0.0072 |
| P97839 | Disks large-associated protein 4 | 0.61 | Down | 0.0056 |
| P20650 | Protein phosphatase 1A | 0.71 | Down | 0.0308 |
| Q75Q39 | Mitochondrial import receptor subunit TOM70 | 0.79 | Down | 0.0303 |
| Q5EB77 | Ras-related protein Rab-18 | 0.73 | Down | 0.0277 |
| P20651 | Serine/threonine-protein phosphatase 2B catalytic subunit beta isoform | 0.6 | Down | 0.0004 |
| Q6AYS7 | Aminoacylase-1A | 0.8 | Down | 0.0136 |
| Q9JI03 | Collagen alpha-1(V) chain | 0.67 | Down | 0.0056 |
| P08699 | Galectin-3 | 0.83 | Down | 0.0046 |
| D4A526 | Leukocyte cell-derived chemotaxin 2 (Predicted) | 0.72 | Down | 0.0487 |
| Q5RKH2 | Galactokinase 1 | 0.74 | Down | 0.0484 |
| Q9R1T3 | Cathepsin Z | 0.68 | Down | 0.0463 |
| P28037 | Cytosolic 10-formyltetrahydrofolate dehydrogenase | 0.65 | Down | 0.0319 |
| 08290 | Asialoglycoprotein receptor 2 | 0.73 | Down | 0.0202 |
| P09650 | Mast cell protease 1 | 0.81 | Down | 0.0352 |
| A0A0G2KAH4 | Protein Dock6 | 0.22 | Down | 0.0008 |
| P35565 | Calnexin | 2.41 | Up | 0.0062 |
| P11884 | "Aldehyde dehydrogenase, mitochondrial " | 0.76 | Down | 0.0073 |
| Q63691 | Monocyte differentiation antigen CD14 | 0.82 | Down | 0.0402 |
| A0A096MJ38 | Protein Ifit1lb | 0.69 | Down | 0.0037 |
|  |  |  |  |  |
