# Supplementary material for: iTRAQ-based proteomic analysis to identify the molecular mechanism of Zhibai Dihuang Granule in the Yin-deficiency-heat syndrome rats
Source: Chin Med. 2018 Jan 8;13:2. doi: 10.1186/s13020-017-0160-y (PMC5759191; doi:10.1186/s13020-017-0160-y)
Supplement: Supplementary file 4 — Additional file 4. STRING analyses of the differentially expressed proteins. [file 13020_2017_160_MOESM4_ESM.docx]

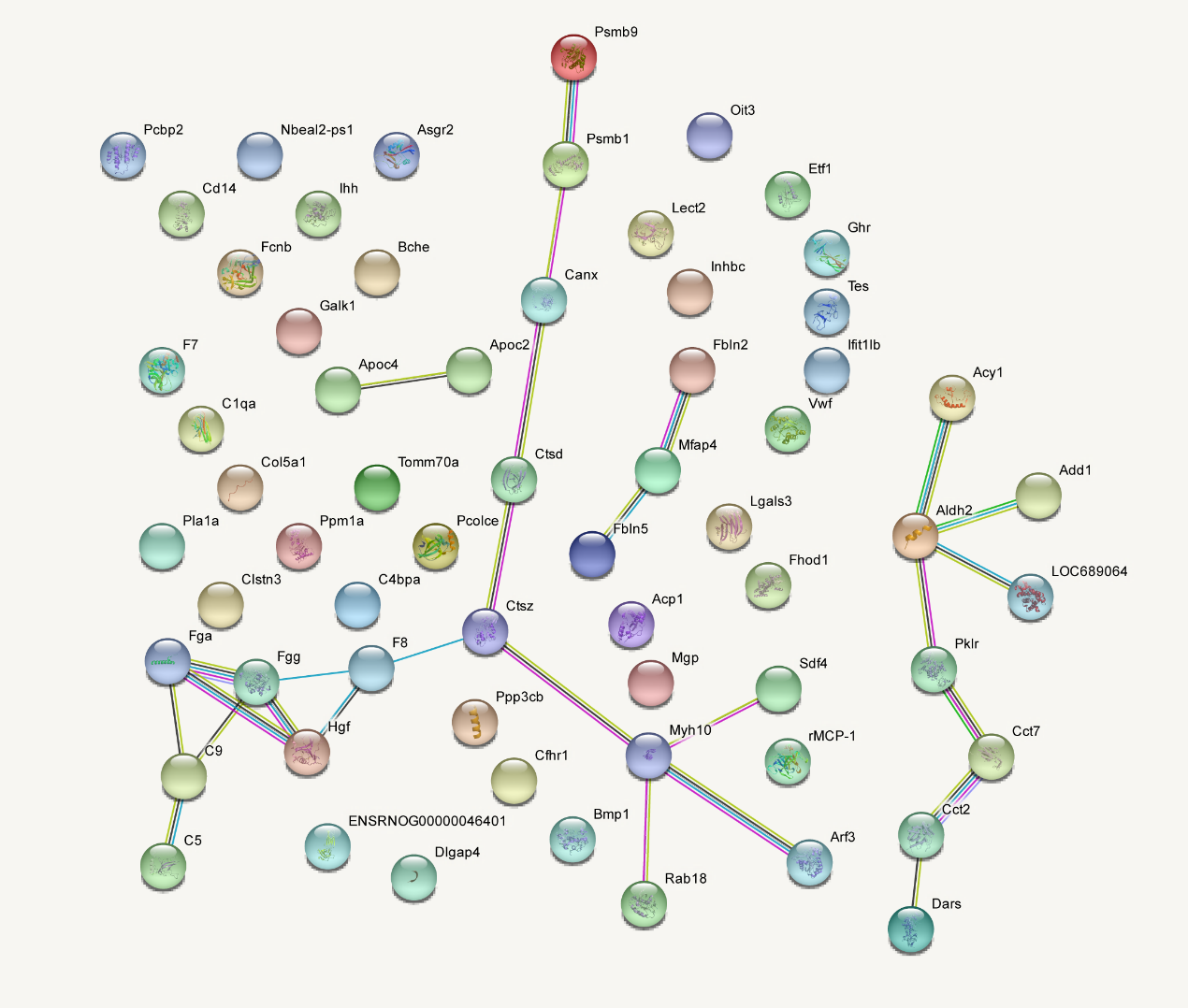


**Figure S1.** Differentially expressed proteins in STRING (Search Tool for the Retrieval of Interacting Genes/Proteins) database.

**Table S2**. Pathway analysis of differentially expressed proteins in STRING database.

| **pathway ID** | **pathway description** | **count in gene set** | **false discovery rate** |
| --- | --- | --- | --- |
| 4610 | Complement and coagulation cascades | 8 | 3.76E-09 |
| 5133 | Pertussis | 4 | 0.00465 |
| 5020 | Prion diseases | 3 | 0.0115 |
| 5150 | Staphylococcus aureus infection | 3 | 0.0196 |
|  |  |  |  |
