# Supplementary material for: iTRAQ-based proteomic analysis to identify the molecular mechanism of Zhibai Dihuang Granule in the Yin-deficiency-heat syndrome rats
Source: Chin Med. 2018 Jan 8;13:2. doi: 10.1186/s13020-017-0160-y (PMC5759191; doi:10.1186/s13020-017-0160-y)
Supplement: Supplementary file 5 — Additional file 5. Quality control validation of MS Data. [file 13020_2017_160_MOESM5_ESM.docx]

QC validation of MS data.

**Figure S2.** Mass error distribution of all identified peptides,

**Repeat1**

**Repeat2**

**Repeat3**

**Figure S3.** Peptide length distribution.

**Repeat1**

**Repeat2**

**Repeat3**

**Figure S4.** **Pearson correlation analysis**

Red represents positive correlation whereas green represents negative correlation. The lighter the tone used, the less significant the corresponding correlation, and white represents a lack of correlation.
